# Thermal stability and topological protection of skyrmions in nanotracks

**DOI:** 10.1038/s41598-017-03391-8

**Published:** 2017-06-22

**Authors:** David Cortés-Ortuño, Weiwei Wang, Marijan Beg, Ryan A. Pepper, Marc-Antonio Bisotti, Rebecca Carey, Mark Vousden, Thomas Kluyver, Ondrej Hovorka, Hans Fangohr

**Affiliations:** 1 Faculty of Engineering and the Environment, University of Southampton, Southampton, SO17 1BJ United Kingdom; 2 Department of Physics, Ningbo University, Ningbo, 315211 China; 3 European XFEL GmbH, Holzkoppel 4, 22869 Schenefeld, Germany

## Abstract

Magnetic skyrmions are hailed as a potential technology for data storage and other data processing devices. However, their stability against thermal fluctuations is an open question that must be answered before skyrmion-based devices can be designed. In this work, we study paths in the energy landscape via which the transition between the skyrmion and the uniform state can occur in interfacial Dzyaloshinskii-Moriya finite-sized systems. We find three mechanisms the system can take in the process of skyrmion nucleation or destruction and identify that the transition facilitated by the boundary has a significantly lower energy barrier than the other energy paths. This clearly demonstrates the lack of the skyrmion topological protection in finite-sized magnetic systems. Overall, the energy barriers of the system under investigation are too small for storage applications at room temperature, but research into device materials, geometry and design may be able to address this.

## Introduction

The current paradigm of magnetic information storage technology, a technique called perpendicular media recording, is coming to a limit where magnetic grains, which are used as information bits, cannot be reduced further in size, since these units lose stability and information can no longer be recorded. New methods for further increases in data storage capacity are desired. One of the main constraints for any data processing and storage technology is the stability of the data, i.e. the robustness of information carriers against random thermal fluctuations at operating temperature.

To analyse the stability of a system we can compute the energy barrier between two of its equilibrium states (that can be used together to represent one bit of information). This barrier is the energy that thermal fluctuations, or any other excitation, needs to provide to the system to drive one equilibrium state to the other. The energy barrier can be used to estimate the average time that the system can remain in each state and thus provides the time scale over which information can be stored in the device without corruption.

Magnetic skyrmions are considered an alternative technology to address the challenges in device engineering^1–5^. Skyrmions are magnetic structures that can be associated with topologically charged particles. They have interesting topological properties that contribute to their stability and they are more energetically efficient to manipulate^1–5^ than magnetic domain walls. Skyrmions arise in systems with a broken symmetry due to the Dzyaloshinskii-Moriya Interactions (DMIs) and can be stabilised using magnetic fields or strong anisotropies in large samples^3, 6, 7^. On the other hand, different works^3, 5, 8–10^ have indicated that small confined systems made of DMI materials are suitable for stabilising skyrmions without the need of an external field.

In the context of possible geometries for the fabrication of a skyrmion based magnetic device, nanotracks have been suggested and a number of studies have shown how a series of skyrmions can be stabilised and driven along the sample by means of weak electric currents^1, 2, 4, 11–13^. In this skyrmion-based racetrack memory design the information bit 0 or 1 would be encoded by a skyrmion’s presence or absence. An important parameter that needs to be understood is the thermal stability. More precisely, we need to explore the energy landscape, find paths which the system can take in the transition between skyrmion and uniform state, and identify the one with the lowest energy barrier. Based on the size of this lowest energy barrier, the lifetime of the binary data can be estimated.

In this study we calculate energy barriers associated with the destruction and creation of skyrmions in thin ferromagnetic nanotracks with interfacial DMIs. The methods can be extended to magnetic solids with different DMI mechanisms and different geometries. Our results are relevant to a variety of interfacial DMI based materials since we analyse these systems in a range of DMI strengths and thus skyrmion sizes. The energy barrier calculations is achieved through a numerical technique called Nudged Elastic Band Method^14–16^ (NEBM), which calculates minimum energy transitions between equilibrium states in magnetic systems. We use an optimised version of the algorithm recently proposed by Bessarab *et al*.^16^ since they claim that early versions of the method^17–20^ lead to uncontrolled behaviour of the algorithm. In particular, we are interested on the minimum energy path between two states, since it gives us the smallest energy required, *i*.*e*. the energy barrier for a configuration to transition to another state.

Our results show that there are three main minimum energy transitions for destroying a skyrmion in a nanotrack within a range of DMI magnitudes. The lowest energy path is one where the boundaries of the system play a major role, making evident the lack of topological protection of the skyrmion in a finite sample. The other two paths with larger energy barriers are a skyrmion collapse and a skyrmion destruction mediated by a singularity. We describe and simulate the system using a (Heisenberg-like) discrete spin model because skyrmion destruction mechanisms that are not mediated by a boundary are forbidden under a continuum description of the magnetisation field due to the skyrmion topology. Hence, estimates of the energy barriers of these energy paths within the micromagnetic model will depend on the numerical discretisation.

Although recent works have been published about skyrmionic systems with interfacial DMI^21, 22^, the key difference is that those works consider large samples, simulating a skyrmion in an infinite system, thus they have not observed the effects of the boundaries, which we identify as the most important route due to the lower energy barrier.

Our discussion will start with a brief introduction to the main concepts of the NEBM and, consequently, the application of the algorithm to nanotracks of different DMI strength using an atomistic simulation framework. Accordingly, we use a hexagonal lattice and we find the three aforementioned skyrmion destruction mechanisms.

## Results

The Nudged Elastic Band Method (NEBM) is an algorithm that searches for minimum energy transitions between two equilibrium states. First results of this numerical method applied to micromagnetics were published by Dittrich *et al*.^17^, computing minimum energy paths (transitions with minimal energy cost) in a variety of simple magnetic systems, which are corroborated with analytic theory. For example, they showed minimum energy transitions of small particles and elongated particles, where paths are characterised by coherent rotations and domain wall propagations, respectively. To help interpret the main results in this work, we need to define some basic terminology.

### Numerical method

**Figure 1:**
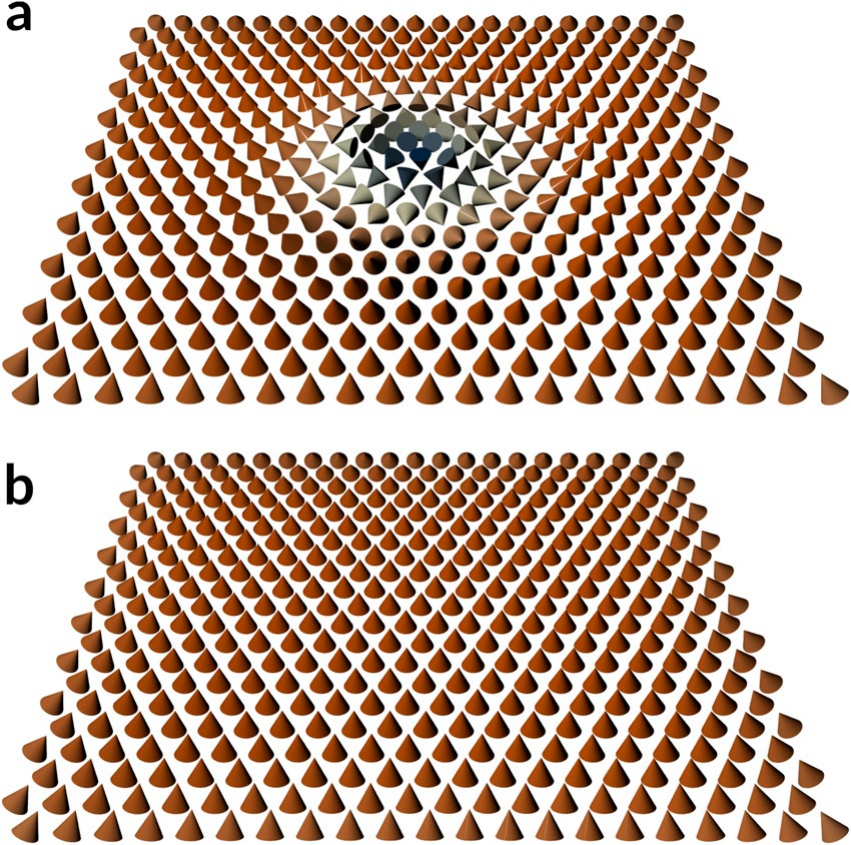
Magnetic configurations in a system with interfacial DMI. Representation of: (**a**) a Néel skyrmion and (**b**) a ferromagnetic ordering in a thin magnetic film with interfacial DMI.

To describe a magnetic material we use a discrete spin model, where we define a lattice of *P* nodes which each have associated a three dimensional spin vector **s**_*i*_, *i* ∈ {0, 1, …, *P* − 1}. This whole system (**s**_0_, **s**_1_, …, **s**_*P*−1_) will be called an *image*, and we will denote it as **Y**. For example, the skyrmion and the uniform state shown in Fig. 1 are one image each. The geometric ordering of the lattice that represents the arrangement of molecules or atoms, is given by crystallographic nature of the material. Depending on the magnetic configuration of the magnetic moments, an image will have a specific energy. Thus, the energy *E* = *E*(**Y**) of the magnetic sample is parametrised by the magnetic ordering of **Y**.

**Figure 2:**
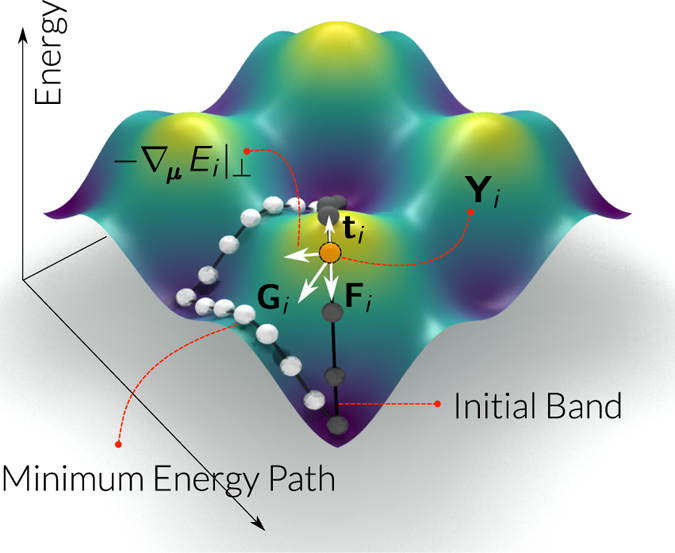
Overview of the NEB method. A summary of the Nudged Elastic Band method in a system parametrised by two variables. The surface height indicates the energy plotted for all points in this two-dimensional phase space. A set of specific magnetic configuration is shown through spheres, each being one image of the method. For a particular image **Y**_*i*_ of the initial energy band, we show the effective (total) force **G**, the gradient component perpendicular to the band **∇**_***μ***_ *E*|_⊥_ (***μ*** = *μ*_s_ **s** as the magnetic moment, see Methods for details), the tangent to the band ***t*** and the spring force **F**. The height of the surface *E*_*i*_ = *E*(**Y**_*i*_) shows the energy of image *i*. At the extrema of the band, the magnetic configurations are fixed and localised at energy minima.

In the NEBM, we define a so called *band* of *N* images **Y**_*i*_, *i* ∈ {0, 1, …, *N* − 1}, which are identical systems in (ideally) different magnetic configurations. For each of the images at either end of the band, **Y**_0_ and **Y**_*N*−1_, we fix the magnetic configuration to be the two equilibrium states for which we want to find the minimum energy transition. For the other *N* − 2 images, (**Y**_1_, **Y**_2_, …, **Y**_*N*−2_), we need to set up an initial sequence of magnetic configurations. A graphical representation of this set up is shown in Fig. 2 where the in-plane coordinates represent the two-dimensional phase space, every sphere *i* represents a particular magnetic configuration **Y**_*i*_ in that phase space, and the surface represents the energy landscape *E*(**Y**). In this figure, the equilibrium states lie in two global minima and the initial band crosses an energy maximum.

Keeping the images **Y**_0_ and **Y**_*N*−1_ fixed, we apply the NEBM algorithm, which iteratively evolves the band to find the lowest energy path between these two (see the minimum energy path in Fig. 2 that passes through a local minimum). This minimisation is achieved by defining an effective force **G** for every image, which depends on vectors **t** that are tangents to the energy band. Additionally, since the energy of the band is minimised, it is necessary to set a spring force **F** between the images in order to keep them equally spaced in the phase space and avoid that the images cluster around the fixed states. Accordingly, to distinguish them, we use a Geodesic *distance* ^16^ which measures the difference of the spins direction between consecutive images (see Methods section).

When the NEBM reaches convergence (see Methods section), the band will ideally pass through a maximum in energy along a single direction in phase space, which is known as a first order saddle point and it determines the energy barrier between the two fixed configurations. This transition path might not be unique and if it is the one with the smallest energy barrier, we call it the minimum energy path. For the final energy band shown in Fig. 2, there are two maxima since the band crosses a metastable state that could be used as an equilibrium configuration, but we can clearly distinguish a single saddle point between every pair of energy minima. In that case the most relevant first order saddle point would be the one with largest energy, which is the barrier that the system needs to climb to get to the other equilibrium state. In general, there is no guarantee that any of the images in the band sits exactly at the saddle point (commonly, there will be images to either side of the saddle point along the band), and thus the energy at the saddle point (and hence the energy barrier) is generally underestimated. To address this problem and improve the accuracy of the estimate, we can push one of the images into that maximum energy position along the path using a variation of the method called the Climbing Image NEBM^16, 23^. This is based on taking the largest energy point from a relaxed band (with the NEBM), redefine the forces applied to this image and then remove the spring force on it (see Methods for details). As a result, this image will try to climb up in energy along the band (while being allowed to decrease its energy in a direction perpendicular to the band).

The NEBM algorithm implemented in our software^24^ has been tested to reproduce basic models proposed by Dittrich *et al*.^17^, giving successful results. In addition, we reproduced the skyrmion annihilation test problem described by Bessarab *et al*.^16, 25^ using an atomistic model for a two dimensional square lattice of atoms with interfacial DMI, which is discussed in the Test Model section of the Supplementary Information.

To explore the suitability of chiral structures for information recording, we have chosen cobalt samples with DMI for our study, based on recent works on these systems^3, 8, 26^. This will also show us another perspective of the claimed topological protection properties of skyrmions in finite systems: in confined geometries the boundaries play a major role on the skyrmion stability. Accordingly, based on ref. 3, we define an 80 nm long, 40 nm wide and 0.4 nm thick stripe, which we discretise into a 320 by 185 spin lattice with a lattice constant of 2.5 Å (see Methods for details). This system has an interfacial DMI whose magnitude we vary and a strong uniaxial out of plane anisotropy. The DMI in a Co based system can be obtained by stacking the cobalt on top of a heavy metal with a larger spin orbit coupling and experimental techniques have been proposed to tune the DMI magnitude^26, 27^. At the time of publication of ref. 3, there was no experimental evidence of the Co samples under study, thus the magnetic parameters are based on standard Co material. Correspondingly, the atomic layer spacing is assumed as *a*_*z *_= 2.5 Å and a lattice constant of *a* = 2.5 Å. The atomic arrangement of an FCC cobalt layer has an hexagonal structure^3, 21, 28^.

Including the strong anisotropy and the confined geometry, the interfacial DMI in a Co layer favours the stabilisation of chiral structures. In general, we observe four well defined equilibrium states, within the range of DMI magnitudes we study. Two of them are Néel skyrmions with the core pointing up or down with respect to the out of plane (*z*) direction (see Fig. 1a) and that are degenerate in energy. Similarly, the two other are degenerate ferromagnetic orderings pointing perpendicular to the nanotrack plane (Fig. 1b), with a small canting at the boundary of the system. We obtained these states by relaxing similar configurations using the Landau-Lifshitz-Gilbert equation. However, we cannot guarantee that these are the only equilibrium configurations since, depending on the *D* value, other chiral orderings can arise but they cannot be easily identified. Generally, knowing the true global minima of a specific system is not straightforward, but we will focus on the four aforementioned magnetic states, using them as fixed NEBM images at the extrema of the energy bands.

### Nanotracks

We begin our analysis by performing a systematic study of long tracks with different DMI constants. We define the DMI in a range from 2.6 up to 3.6 mJ m^−2^ in steps of 0.2 mJ m^−2^. To apply these values to a discrete spin model, we converted them from micromagnetic values considering the hexagonal nature of the sample. Therefore, the equivalent atomistic DMI constants in the discrete model range from *D* = 0.586 meV to 0.811 meV in steps of 0.045 meV.

In the range of DMI magnitudes we choose, the skyrmion energy is always larger than the uniform configuration energy, but it starts to get closer to that of the ferromagnetic state as the *D* value increases. Additionally, the systems differ in skyrmion size, where a skyrmion gets larger as the DMI constant increases (see Supplementary Fig. S2 for detailed values of the skyrmion sizes). The length of the track is not relevant as long as the skyrmion size is not affected by the long edge boundaries, since the isolated skyrmion does not interact with them. On the other hand, the stripe width is defined according to ref. 3, which is reasonable for a novel technological application, and the skyrmion will slightly interact with the short edge boundaries when the DMI is strong enough. Larger DMI magnitudes than the values we specify are not analysed since skyrmions acquire an elongated shape^3, 29^ and the skyrmion loses its symmetrical character. A different method to modify the skyrmion dimensions, rather than varying the DMI, is tuning the uniaxial anisotropy, where a stronger anisotropy reduces the skyrmion size. However its mechanism is different to the antisymmetric exchange, thus the energy landscape is likely to change and analysing this effect goes beyond the scope of this study.

For every case, we use two different initial bands for the NEBM: (i) a linear interpolation using spherical coordinates, which means interpolating the spherical angles that describe the magnetic moments and (ii) a skyrmion displacement towards one edge of the disk, making it disappear at the boundary.

After relaxation with the NEBM, we obtained three different transitions. One of these paths is a symmetric skyrmion collapse (shrinking) until the last spin at the skyrmion centre flips to give rise to the ferromagnetic ordering, which originates from the linear interpolation initial state. The second transition is given by the annihilation of the skyrmion core through a singularity that resembles a Bloch point, and this is observed for DMI magnitudes of 0.676 meV and above. The third transition we observe is given by the displacement of the skyrmion towards the boundary, where the skyrmion configuration is deformed until annihilation. Detailed images of these three different transitions and the initial states are provided in Supplementary Figs S3 and S4.

### Boundary annihilation

**Figure 3:**
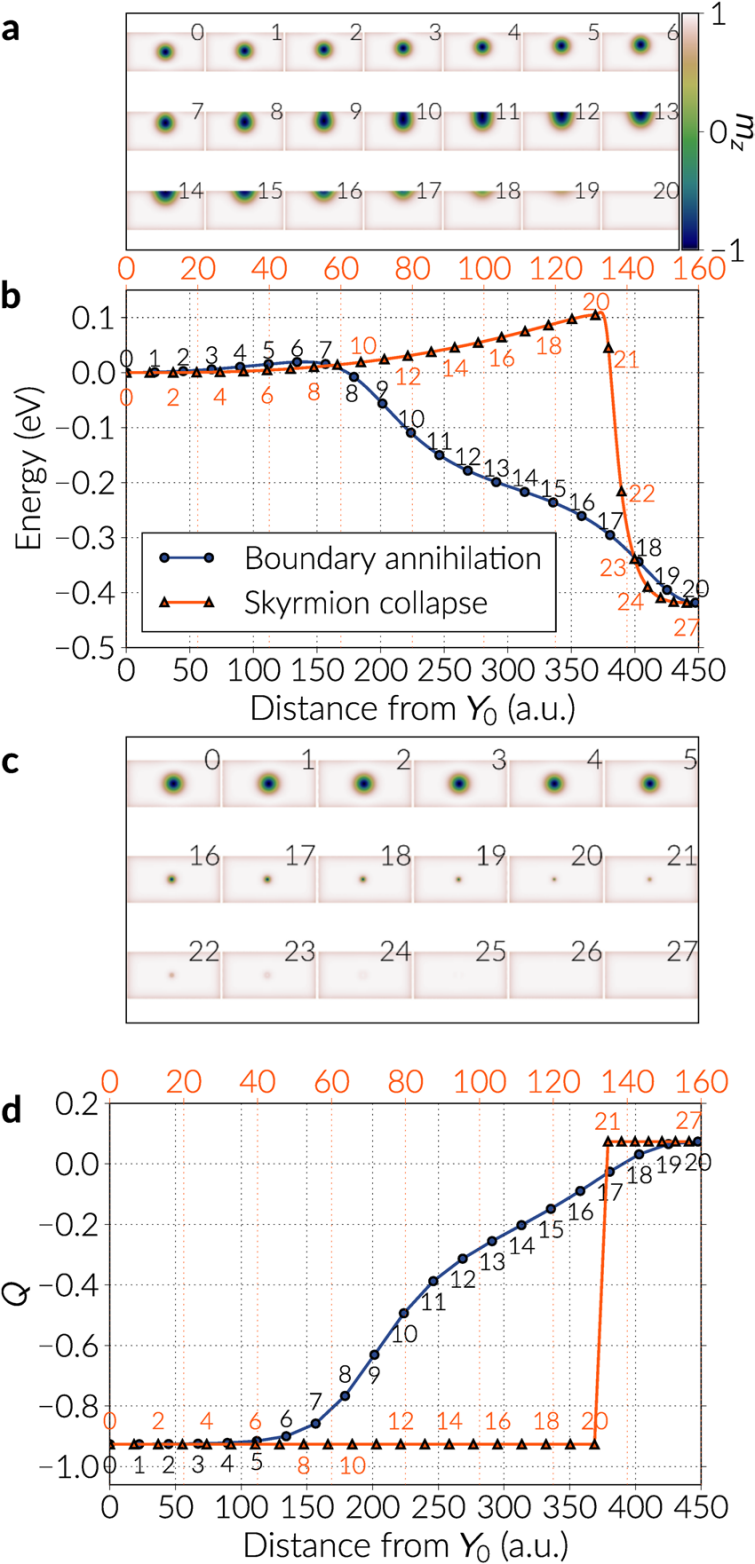
Minimum energy paths of a skyrmion in a cobalt nanotrack. The DMI constant of the system is *D* = 0.676 meV of magnitude. There are two different paths: a skyrmion annihilation at a boundary and a symmetrical skyrmion collapse. (**a**) Images of the band for the boundary annihilation, annotated according to the numbers in the corresponding curve in (**b**). The colour scale refers to the out of plane (*z*) component of the magnetisation field. (**b**) Energy bands for both minimum energy paths as a function of the distance from the first image (left extreme of the bands). The top scale refers to the skyrmion collapse case. (**c**) Images of the band for the skyrmion collapse. (**d**) Topological charge (skyrmion number) *Q* as a function of the images distances for the cases depicted in (b). The top scale refers to the skyrmion collapse case.

We observe the skyrmion annihilation at a boundary for every DMI value. In particular, for the case of *D* = 0.676 meV, we show in Fig. 3b the energy band for this path and in Fig. 3a, the corresponding images where the skyrmion is annihilated at the boundary (see also Supplementary Video S4). Additionally, in Fig. 3d, we illustrate the total topological charge of the images where a smooth transition towards the uniform state occurs.

**Figure 4:**
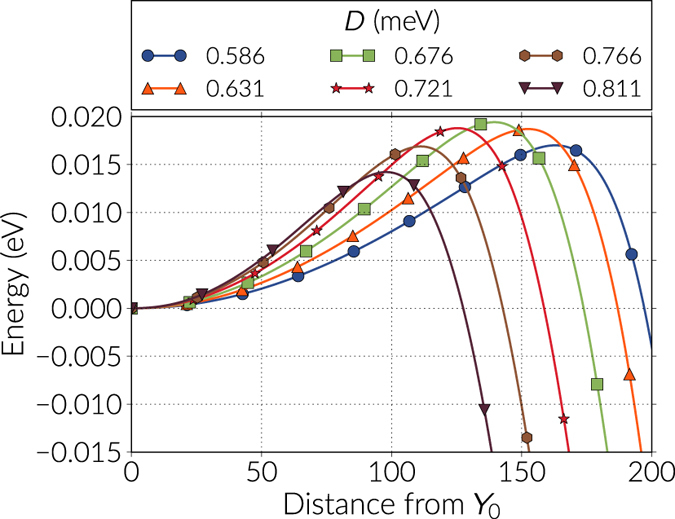
Energy bands for the skyrmion annihilation at the boundary. The bands are shown for different DMI constant *D* and only for the first few images of the bands. The first or extreme left image is the skyrmion configuration and, for every case, the energy is redefined with respect to the skyrmion energy. The continuous line is a cubic polynomial interpolation.

**Figure 5:**
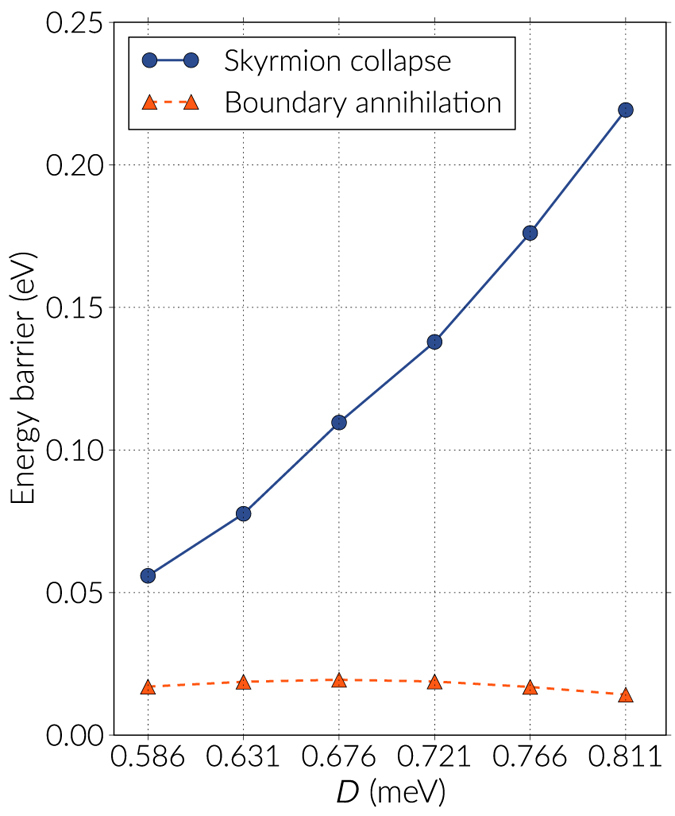
Energy barriers for two different energy paths. The energy barrier dependence on the DMI constant is shown for skyrmion collapse and skyrmion annihilation at the boundary. The energy barriers are calculated with respect to the skyrmion energy and using a cubic polynomial interpolation on the images of the energy bands.

We analyse the dependence of the energy bands with respect to the DMI strength in Fig. 4 for the first few images of every band, and in Fig. 5 we plot the energy barriers with respect to the skyrmion energy using the continuous curve approximation, which is a cubic polynomial that uses information from the tangents of the images in the band (a comparison of the energy barriers values using the approximation with respect to the actual data points is shown in the Supplementary Information). The tendency in Fig. 5 is that the barriers decrease almost quadratically with larger DMI magnitudes, where a maximum is observed around *D* = 0.676 meV (see Table 1). In general, these barriers are significantly smaller than the energy difference between the skyrmion and the ferromagnetic state. To check the robustness of the results we also modified the NEBM spring constant values and we found out that there are small variations of the barriers when changing this parameter, which is mostly due to the image positioning in the band, and if the spring constant is too large the method struggles to converge. In the results of Fig. 4, we can notice that when increasing the DMI strength, the resolution of the images before the saddle point gets poorer, however these variations are not large and applying the climbing image method to the largest energy states gives energy barrier magnitudes similar to those obtained when performing a polynomial approximation of the band.

**Table 1:** Energy barriers for the skyrmion destruction energy paths. Energy values are computed with respect to the skyrmion energy for different DMI constants. The singularity driven skyrmion destruction is only observed for strong enough DMI constant magnitudes.

*D* (meV)	Energy barrier (eV)		
	Boundary	Collapse	Singularity
0.586	0.0170	0.0559	
0.631	0.0187	0.0776	
0.676	0.0194	0.1096	
0.721	0.0188	0.1379	0.3445
0.766	0.0169	0.1761	0.3728
0.811	0.0142	0.2193	0.4016

In the range of DMI magnitudes we analysed the skyrmion destruction at the boundary is the transition that has the lowest activation energy. Specifically, the energy barriers are an order of magnitude smaller than the skyrmion collapse transitions. This demonstrates the lack of topological protection for the skyrmions, which is due to the finiteness of the system.

### Skyrmion destruction

When relaxing energy paths that directly involve the destruction of the skyrmion core in a region inside the track, the spins at the center of the skyrmion reverse to form a ferromagnetic state. This is a non trivial process since the band suffers drastic changes in energy when the NEBM tries to converge to a minimum energy transition and the system must undergo a topological change. This path is (according to our observations) the most likely for a skyrmion situated in a large or infinite sample, where it is meant to be topologically protected, thus we expect a larger energy barrier than in the transition mediated by a boundary.

**Figure 6:**
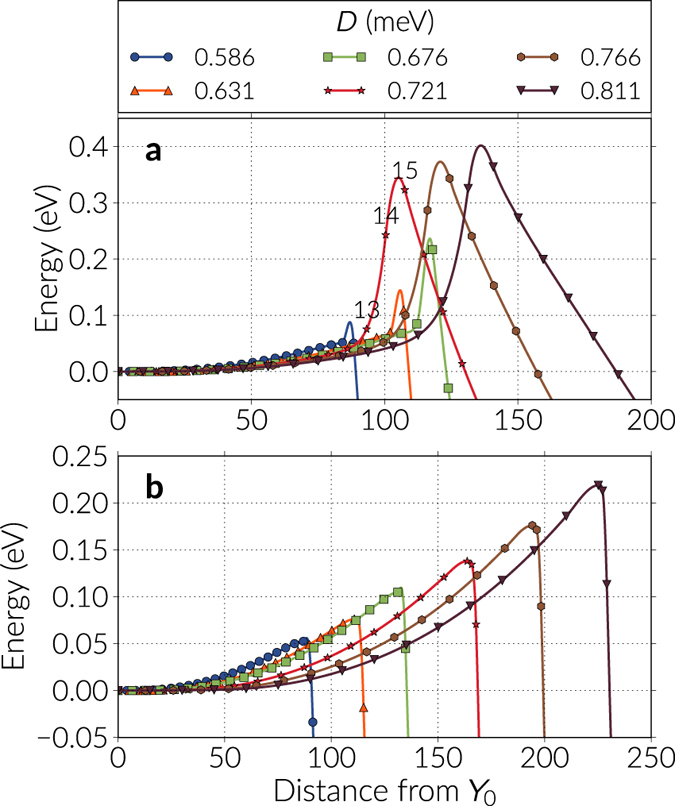
Energy bands for two skyrmion destruction mechanisms. The bands are shown for different DMI constant *D* and only for the first few images of the bands. The first or extreme left image is the skyrmion configuration and for every case, the energy is redefined with respect to the skyrmion energy. The continuous line is a cubic polynomial interpolation. (**a**) The energy bands obtained with the NEBM. The curves for DMI values of *D* = 0.676 meV and below, are the skyrmion collapse. For values of *D* = 0.721 meV and above, the bands are the skyrmion destroyed by a Bloch point like singularity. (**b**) Refined energy bands obtained with the Climbing Image NEBM applied to the largest energy points of the curves in Figure (**a**). We expect the data in (**b**) to be a better approximation of the energy barrier. For this case, all the bands converged towards the skyrmion collapse path.

We firstly found that for DMI magnitudes of *D* = 0.676 meV and below, the algorithm converged to the skyrmion collapse process, which is similar to the one depicted in Fig. 3c. In Fig. 6a we show the first images of the bands, where the skyrmion state is given by the left extrema, and we observe pronounced peaks at the saddle points. These points are the images that have a tiny skyrmion with only a few spins defining its core before reversing. It is worth noting that these saddle points have a finite energy since we have a discrete number of magnetic moments, whereas in a continuum model it is likely that the peak depends on the discretisation of the continuum mesh that defines the material. In our results, the saddle point energies (and thus the energy barriers) increase with the DMI constant, where values range between 0.1 and 0.25 eV larger than the skyrmion energy. Around the saddle points, the energy landscape must have a rough shape since the neighbouring images usually have a substantial change in energy. Specifically, we observed that when the images move along the band before the algorithm reaches convergence, the images that cross this region suffer large energy alterations (see Supplementary Video S1 as an example). In order to resolve more accurately the energy value of the saddle points, we applied the climbing image technique on them, slightly reducing their energy and improving the resolution around them. We show these results in Fig. 6b (see also Supplementary Video S5). The value of the energy barriers are summarised in Fig. 5 and Table 1, where the magnitudes increase almost linearly with the DMI magnitudes. Furthermore, as we did for the boundary annihilation, using the case of *D* = 0.676 meV, we plot in Fig. 3b the energy band of the skyrmion collapse path and the corresponding images in Fig. 3c. We notice that the saddle point lies between the 20th and the 21st image, where the last few spins at the tiny skyrmion core reverse, and there is a drastic change in energy in the next images. A different perspective of this phenomenon is observed when looking at the topological charge values of the images in Fig. 3d. Up to the 20th image, the images preserve the skyrmion structure, thus having equivalent skyrmion number. However, after this point the skyrmion core is reversed and the skyrmion ordering is lost, which is indicated by a sharp change in the topological charge magnitude.

**Figure 7:**
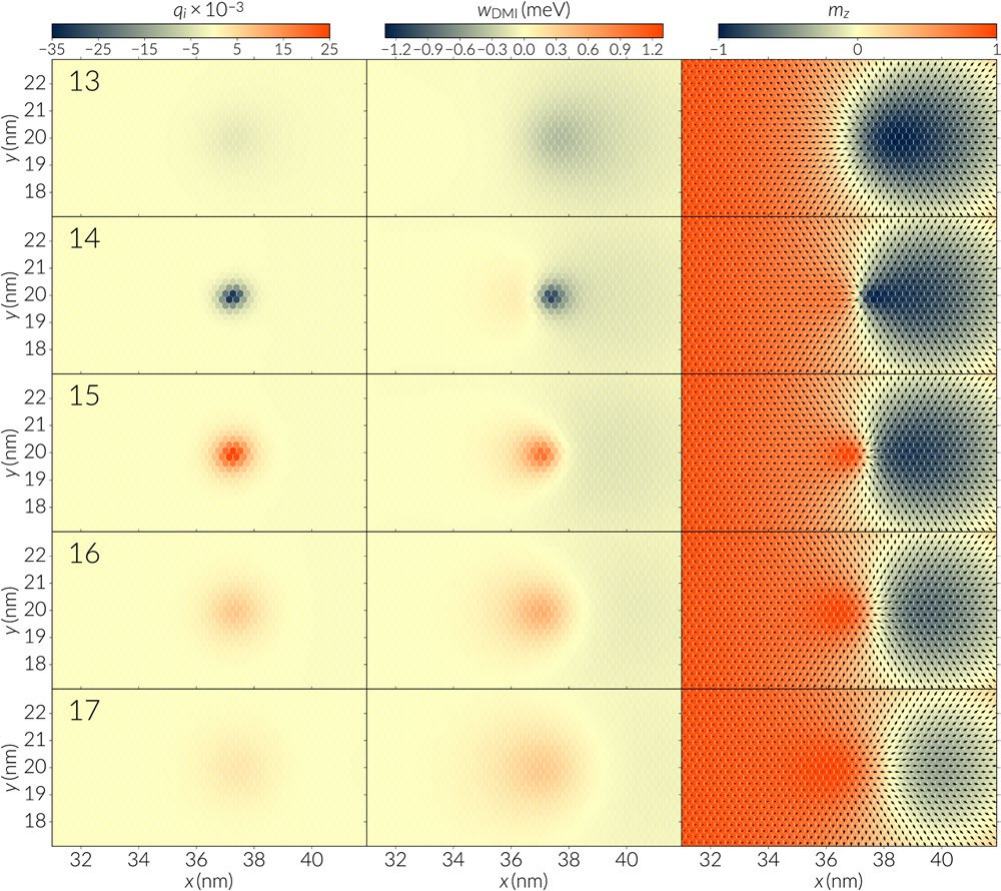
Skyrmion destruction mediated by a topological singularity. The sequence shows the snapshots of the energy band images for an nanotrack system of DMI constant *D* = 0.721 meV, described by a hexagonally arranged discrete spin lattice. This band corresponds to the annotated curve of Fig. 6a. The left column shows the topological charge density, the middle sequence the DMI energy density per lattice site and the right column the corresponding magnetisation fields. The hexagonal mesh can be distinguished from the honeycomb shaped data points. The numbers at the top left of every row indicate the image number from the energy band.

For DMI magnitudes of *D* = 0.766 meV and above, the NEBM converged to a path where the skyrmion core is destroyed by the emergence of a singularity close to the skyrmion boundary. These paths have associated energy barriers of above 0.3eV (see Table 1) and we show the first images of the bands in Fig. 6a (see Supplementary Video S2 for the evolution of the band and Supplementary Video S6 for the transition). The singularity that drives the skyrmion towards the ferromagnetic state resembles a Bloch point, and Fig. 7 shows the sequence of images of the band where this structure destroys the skyrmion, for the system with a DMI constant of *D* = 0.721 meV. This series of snapshots are zoomed around the skyrmion core and the numbers at the top left of every row indicate the image numbers in the band, which correspond to the annotated points of the curve with stars in Fig. 6a. The left column in Fig. 7 is the topological charge per lattice site *q*_*i*_, the middle column is the DMI energy density and the right column shows the spin vector field where the colors indicate the component of the magnetisation perpendicular to the track plane, *m*_*z*_. At the 14th image of the sequence (second row), we observe that the skyrmion core concentrates to the left side of the original skyrmion core with a drop in negative DMI energy. Moreover, the topological number is reduced since the spins in a tiny region cover most of the directions in a unit sphere, like a small skyrmion (at the 13th snapshot the topological charges are smaller since the different spin directions are more spread out). Consequently, at the 15th image a singularity emerges, which has a chirality opposite to the skyrmion, indicated by the positive gain in DMI energy and a positive charge. At this point, the skyrmion core has already been annihilated. The singularity has a hedgehog structure occupying a radius of around 3 lattice sites, and it will expand to give rise to the ferromagnetic state at the end of the energy band, decreasing the DMI energy. Characterising this singularity as a Bloch point is not trivial due to its two dimensional nature and the lacking of an appropriate resolution to mathematically define it. In fact, the critical process lies between the 14th and 15th image and if we refer at Fig. 6a, the 15th image is close to the saddle point of the band. Interestingly, diverse studies^3, 30, 31^ have reported the mechanism of destroying or nucleating a skyrmion by means of a non trivial topological structure, when applying spin polarised currents. In particular, Elías and Verga^30^ characterise this singularity by its internal magnetic field, which relates to the topological charge, and Sampaio *et al*.^3^ show how a singularity concentrates DMI energy at the border of the skyrmion before it is nucleated (see Fig. S3 of their Supplementary Information). This transition is not a minimum energy path but it is an alternative path to disrupt the skyrmion stability that seems to be preferred by the system when following certain dynamical processes.

Although the bands where a singularity destroys the skyrmion converged without exhibiting the energy fluctuations observed in the cases with smaller DMI constants (see Supplementary Fig. S5), they are not very stable and are likely to pass through a higher order saddle point. This is because after applying the climbing technique on the images at the saddle points, the bands converged towards the skyrmion collapse transitions. These paths have smaller energy barriers, as shown in Fig. 6b, and the energy barrier has an approximately linear dependence on the DMI magnitude, as mentioned previously.

Regarding the simulations with a climbing image, it is usually the case that this variation of the algorithm helps the saddle point to climb up in energy along the band and thus improve the value of the energy barriers^16, 23^. However, for the skyrmion simulations, the largest energy points decreased their energy, as seen in Fig. 6. Furthermore, we analysed the evolution in energy of these saddle points with respect to the number of iterations of the algorithm, and we saw that they oscillate for a while before reaching a more stable state (see Supplementary Fig. S5 and Supplementary Video S3), which is given by the symmetrical destruction of the skyrmion, and these oscillations are prolonged for systems with larger DMI magnitudes. A plausible explanation for this phenomenon is as follows. As we mentioned before, the energy landscape has a rough shape or small peaks in energy around a critical point. In particular, this must be caused because a singularity that destroys a skyrmion could appear in any place around the skyrmion boundary, giving multiple possibilities for a saddle point. Additionally, besides a component of the effective force that makes a climbing image to go up in energy along the band, there is also a component that still allows it to follow a direction of minimum energy. Therefore, as the image tries to climb, it may also be pushed to a small minimum energy region if it is over a very narrow peak in the landscape, decreasing its energy. Through these dynamics, the climbing image can, overall, move to a region of lower energy and possibly to a smoother energy region, where it finally settles. Thus, according to our results, the climbing image is a mechanism that helps us to find more equilibrium solutions for the minimum energy transitions.

## Discussion

Studying the paths through phase space along which a skyrmion can be annihilated from a track or created in the track, we find that for the geometry studied the lowest energy barrier is generally found by using the boundary of the track (see transition snapshots in Fig. 3a). This transition path circumvents the topological protection, as has been pointed out by Streubel *et al*.^32^ explaining that the skyrmion topological protection is non-existent due to the system finiteness. Therefore, the low stability of these configurations found by the NEBM simulations seems reasonable. We quantify this stability by analysing the results using the Arrhenius-Néel law for the relaxation time

1 $$\tau ={\tau }_{0}\exp (\frac{{\rm{\Delta }}E}{{k}_{{\rm{B}}}T})$$

where *τ*_0_ is related to the attempt frequency *f*_0_ = *τ*_0_^−1^, *T* is the temperature and *k*_B_ is the Boltzmann constant. The attempt frequency magnitude is not easy to obtain since the theory normally refers to simple or macrospin systems, but its value is usually in the range^33, 34^ 10^9^ to 10^12^ Hz. Using our computed values for the skyrmion energy barriers and *f*_0_ = 10^9^ Hz, we obtain, at room temperature: (i) for the boundary annihilation in nanotracks with *D* = 0.676 meV (barrier of approximately 0.0194 eV) an average lifetime of *τ* = 2.117 ns, whereas (ii) for the skyrmion collapse, the barrier is 0.110 eV, thus this gives us *τ* = 70.347 ns. We must notice that the exponential is very sensitive to the energy barrier values, which can make a difference if the system follows the skyrmion collapse path, and *f*_0_ could be smaller, which would make the lifetimes some orders of magnitudes larger. Although these times seem very small, they are computed at room temperature, hence an experiment^35^ at 4.2 K, for example, would make a relaxation time of *τ* = 1.864 × 10^14^ s. To confirm the phenomenon of low barriers, it would be ideal to compare experimental data showing skyrmion switching times that could be used to estimate an attempt frequency and exponential law for its average lifetime.

Moreover, we can compare our calculated energy barriers with respect to recent magnetic recording technology. In the context of heat assisted magnetic recording technology, using the data from Weller *et al*.^36^ and approximating a magnetic grain with a spherical shape, the energy barrier for that system is approximately 95.54 *k*_B_ *T* at room temperature (*T* = 300 K). In addition, from typical parameters of magnetic grains in perpendicular recording media, provided by Richter^37^, we can estimate energy barriers of about 97.06 *k*_B_ *T*. On the other hand, for the skyrmionic system of *D* = 0.676 meV, the boundary annihilation gives us a barrier of only 0.75 *k*_B_ *T* and the skyrmion collapse a barrier of 4.24 *k*_B_ *T*, which substantiates our findings of low thermal stability of skyrmions in confined systems.

This phenomenon of low stability must also be present in other finite geometries, like cylindrical structures that have been proposed recently^3, 9, 10^, complicating any technological application of these systems, at least at room temperature, since at lower temperatures the energy barrier can be significant. For skyrmion based technology to operate at room temperatures, we need to find systems with larger energy barriers for skyrmion destruction; other DMI hosting materials such as those with a bulk interaction, or those of increased thickness may have larger barriers. This claim is only speculative since modifying the parameters involved in the system, such as adding thickness to the sample or applying external magnetic fields, changes the energy landscape and different paths would be encountered with the NEBM relaxation, which would require a proper analysis in a different study. On the other hand, a recent theoretical study^38^ has proposed taking advantage of edge instabilities for the creation of skyrmions through the boundaries of a track, avoiding other larger topological energy barriers from different energy paths.

The processes of destroying the skyrmion by a collapse or by a singularity, are energetically feasible due to the discrete nature of the crystal lattice. In the case of an infinite system (i.e. no geometry boundaries), we have observed so far that these paths are the only possible ways to destroy a skyrmion. Thus having a skyrmion far away from boundaries is an option to get more stability but requires larger structures. Regarding the Bloch point like transition, we mentioned that it this not observed for the cases with weaker DMIs. We believe this is because it might be difficult to resolve this structure, which spans a circle of about 3 lattice points of diameter, when the skyrmion is sufficiently small. Our preliminary results on samples of Fe on Ir, where skyrmions are only a few nanometres wide, have confirmed this.

We must emphasize that we are using an atomistic spin model, where each spin represents one atomic magnetic moment and which requires more computational effort than a micromagnetic model. However, a continuum description of the magnetisation field is inaccurate since the skyrmion collapse or Bloch point occurring in the higher energy paths we find, break the assumption of micromagnetics that the magnetisation changes slowly as a function of position. Because of this violation, it is not possible to predict quantitatively the energy associated to the saddle points of these paths, giving the energy barriers dependence on the sample discretisation.

Furthermore, for the skyrmion collapse path the energy barriers are larger for increasing DMI values. This is because the energy of helicoidal configurations approaches that of the ferromagnetic state, reaching a point where helicoids become the ground state of the system. Additionally, larger DMI magnitudes favour multiple twistings of the magnetisation. Therefore, paths that directly involve helicoidal structures are significantly preferred. This is also the reason why the boundary annihilation has very low barriers for strong DMI cases.

For DMI magnitudes below the range we have studied, the energy barriers become smaller and we noticed that at *D* = 0.450 meV the skyrmion collapse has a slightly smaller energy barrier than the boundary annihilation, which is expected from the tendency of the curves of Fig. 5. However, this DMI value is close to a critical value of *D* = 0.405 meV, below which a skyrmion cannot be stabilised anymore. These results are shown in the Supplementary Information.

A recent publication^21^ reports a study on cobalt monolayers by means of the NEBM and two different observed transition paths. Their Path 1 is similar to the skyrmion collapse we have reported in this work and, recently, by Lobanov *et al*.^22^. Their Path 2 adds a rotation of the spins before the core collapses, which resembles a horizontal cut of a three dimensional Bloch point. Although we observe this topological structure, in our case it usually appears at the skyrmion core boundary for large enough skyrmion sizes, which depend on the DMI magnitude. We have only observed that a skyrmion collapse with rotating spins appears as the initial state when we set up the initial band using linear interpolations, but which disappear as the elastic band reduces its energy. This has also been confirmed by Lobanov *et al*.^22^. In this context, a key difference between this skyrmion stability study and refs 21 and 22 is that we take the role of the boundary into account. We find that skyrmion annihilation and creation via the boundary has an order of magnitude lower energy barrier for this track geometry and will thus be the preferred path for the system.

In summary, it is important to consider that due to the finite nature of magnetic samples in real life, skyrmions will have a weaker stability since they can be destroyed through the boundaries and there is no topological protection. It may be possible to overcome this through geometry, material and device design.

All data from this study, used to create the figures, can be reproduced from a repository in ref. 39.

## Methods

### Material specifications

Our main study is focused on thin cobalt nanotracks with interfacial DMI. In these systems, skyrmions are stabilised with the help of an anisotropy perpendicular to the disk or stripe plane. Within a discrete spin model, the Hamiltonian for a cobalt system of *P* atomic sites is described as

2 $$\begin{array}{rcl}H & = & -\sum _{\langle i,j\rangle }^{P}{J}_{ij}{{\bf{s}}}_{i}\cdot {{\bf{s}}}_{j}+\sum _{\langle i,j\rangle }^{P}{{\bf{D}}}_{ij}\cdot [{{\bf{s}}}_{i}\times {{\bf{s}}}_{j}]-\sum _{i}^{P}{{\mathscr{K}}}_{{\rm{u}}}{({{\bf{s}}}_{i}\cdot \hat{{\bf{z}}})}^{2}\\  &  & +\frac{{\mu }_{0}{\mu }_{{\rm{s}}}^{2}}{4\pi }\sum _{\langle i,j\rangle }^{P}[\frac{{{\bf{s}}}_{i}\cdot {{\bf{s}}}_{j}}{{r}_{ij}^{3}}-\frac{3({{\bf{s}}}_{i}\cdot {\hat{{\bf{r}}}}_{ij})({{\bf{s}}}_{j}\cdot {\hat{{\bf{r}}}}_{ij})}{{r}_{ij}^{5}}]\end{array}$$

where the normalised vector **s**_*i*_ is the spin direction at the *i* th site, *J*_*ij*_ and **D**_*ij*_ are the atomistic exchange and DMI tensors between the spin at the *i* − th site with the *j* − th nearest neighbour respectively, which have integrated the *S* ^2^ factor, *S* being the total average spin per lattice site. For an interfacial DMI, we can write the Dzyaloshinskii vector as^28^ ${{\bf{D}}}_{ij}=D{\hat{{\bf{r}}}}_{ij}\times \hat{{\bf{z}}}$. Moreover, ${{\mathscr{K}}}_{{\rm{u}}}$ is the anisotropy constant per lattice site, *μ*_s_ = *gμ*_B_ *S* is the magnetic moment with *g* as the Landé *g*-factor and *μ*_B_ the Bohr magneton, and *r*_*ij*_ = |**r**_i_ − **r**_*j*_| is the distance between two lattices sites, with **r**_*i*_ the position vector of the *i* th spin. The summations with the restriction 〈*i*,*j*〉 means counting pair of spins only once.

In the continuum, the DMI can be theoretically described by the energy density in terms of two Lifshitz invariants. Following the formalism of Rohart and Thiaville^10^ the invariants are defined with an opposite chirality^3^:

3 $${w}_{{\rm{DM}}}=-{D}_{{\rm{c}}}({ {\mathcal L} }_{xz}^{(x)}+{ {\mathcal L} }_{yz}^{(y)}).$$

where *D*_c_ > 0 is the DMI constant. Therefore, for a hexagonal lattice, we use the following relations to convert the micromagnetic parameters into atomistic values

4 $$A=\frac{\sqrt{3}{J}_{ij}}{2{a}_{z}},{D}_{{\rm{c}}}=\frac{\sqrt{3}D}{a{a}_{z}},{M}_{{\rm{s}}}=\frac{g{\mu }_{B}S}{\frac{\sqrt{3}}{2}{a}^{2}{a}_{z}},{K}_{{\rm{u}}}=\frac{{{\mathscr{K}}}_{{\rm{u}}}}{\frac{\sqrt{3}}{2}{a}^{2}{a}_{z}}$$

where *a* is the lattice constant in the atomic layer plane, *a*_*z*_ is the interlayer spacing, *A* is the exchange constant, *M*_s_ the saturation magnetisation and *K*_u_ the anisotropy constant. Using these formulas, we notice that the skyrmion size depends on the dipolar interactions and our atomistic simulations show larger skyrmions than in the continuum model. The standard derivation of the demagnetising field from a discrete model in micromagnetics is based on Brown’s approximations under Lorentz assumptions where, for linearly changing magnetisation fields (or symmetrical square lattices), there is an anisotropic term that is usually not taken into account since it averages to zero^34, 40^. The nonlinearity of the skyrmion structure probably falls outside these approximations, causing the phenomenon we have observed. This issue has been previously mentioned in the literature^41, 42^. To make the atomistic results comparable to the micromagnetic ones^3^, we assume atomistic lattice distances with equal magnitudes along the plane and layer thickness, *i*.*e*. *a* = *a*_*z*_ = 2.5 Å (rather than *a*_*z*_ = 4 Å), obtaining good agreement in skyrmion dimensions for the discrete model. Accordingly, based on the micromagnetic parameters specified on ref. 3 and using equations 4, the atomistic magnetic parameters are *μ*_s_ = 0.846*μ*_B_, *J*_*ij*_ = 27.026 meV and ${{\mathscr{K}}}_{{\rm{u}}}=0.0676\,{\rm{meV}}$. To match the track system of 80 by 40 nm, we specified a lattice of 320 × 185 atoms.

### Nudged Elastic Band Method

Continuing the discussion of the NEBM section, the evolution of the energy bands are made using a first order differential equation where every image is evolved with a fictional time *τ*. In Cartesian coordinates we use a LLG kind of equation, based on the work of Suess *et al*.^18^:

5 $$\frac{\partial {{\bf{Y}}}_{i}}{\partial \tau }=-{{\bf{Y}}}_{i}\times {{\bf{Y}}}_{i}\times {{\bf{G}}}_{i}+c\sqrt{{(\frac{\partial {{\bf{Y}}}_{i}}{\partial \tau })}^{2}}(1-{{\bf{Y}}}_{i}^{2}){{\bf{Y}}}_{i}$$

In equation 5, the last term is to constrain the length of the spins using a suitable factor *c*. The vector **G**(**Y**_*i*_) = **G**_*i*_ is defined as a force (for every image) perpendicular to the band plus a spring force parallel to the band (see refs 14 and 17)

6 $${{\bf{G}}}_{i}=-{\nabla }_{{\boldsymbol{\mu }}}E({{\bf{Y}}}_{i}{)|}_{\perp }+{\bf{F}}({{\bf{Y}}}_{i}{)|}_{\parallel }$$

The gradient in this definition is with respect to the spin moment ***μ*** = *μ*_s_ **s**, which gives the number of degrees of freedom, hence we take advantage of the effective field definition when evaluating the gradient: ${{\rm{\nabla }}}_{{\boldsymbol{\mu }}}E({{\bf{Y}}}_{i})={\mu }_{{\rm{s}}}^{-1}{\rm{\partial }}E/{\rm{\partial }}{\bf{s}}=-{{\bf{H}}}_{{\rm{e}}{\rm{f}}{\rm{f}}}$. The parallel component means following the direction of a tangent vector **t**_*i*_ of an image **Y**_*i*_ which depends on the energy of its neighbours^14, 17^. In the Climbing Image technique, we redefine the **G** vector for a single image (the climbing image) of the energy band, which is usually close to a saddle point, as^23^

7 $${{\bf{G}}}_{i}^{{\rm{CI}}}=-{\nabla }_{{\boldsymbol{\mu }}}E({{\bf{Y}}}_{i}{)|}_{\perp }+{\nabla }_{{\boldsymbol{\mu }}}E({{\bf{Y}}}_{i}{)|}_{\parallel }$$

When evolving the system using equation 5, the tangents **t** and effective force **G** have been projected into the tangent space to avoid misbehaviour of the band when the forces overlap, as specified in ref. 16.

The spring force is defined using the images distance

8 $${\bf{F}}({{\bf{Y}}}_{i}{)|}_{\parallel }=k(|{{\bf{Y}}}_{i+1}-{{\bf{Y}}}_{i}|-|{{\bf{Y}}}_{i}-{{\bf{Y}}}_{i-1}|){{\bf{t}}}_{i}.$$

Correspondingly, the images distances are defined by a Geodesic length using Vincenty’s formulae^16^.

Most of the figures have the abscissa defined as as the distance from the first image of the band **Y**_0_. This simply means summing up the distances from neighbouring images, *i*.*e*. the *i* th image in the band will be at a distance

9 $$d=\sum _{j=0}^{i-1}|{{\bf{Y}}}_{j+1}-{{\bf{Y}}}_{j}|$$

from the first extreme of the band, which we measure in arbitrary units.

We describe the magnetisation using Cartesian coordinates, although any suitable coordinate system could also be used^16^. Our approach to determine the minimum energy band is to set an initial energy band using a linear interpolation of the spin angles between the fixed equilibrium images **Y**_0_ and **Y**_*N*−1_ in spherical coordinates, and then to evolve the band in the chosen Cartesian coordinates to find the minimum energy transition path between the fixed images. The advantages of using a Cartesian description of the spins is that the energy band is better defined when the spins directions are close to the poles but it is still necessary to constrain their length, which is fixed at zero temperature. While we discuss the initial energy band, we note that is also possible to manually specify an initial guess for the transition, usually by taking one or more images from a known path, for example the intermediate states when applying a spin polarised current between two equilibrium states.

We note that with the NEBM one has to hope that chosen initial paths capture the physically relevant transition paths with the lowest energy barriers when minimised, but this cannot be proven. There are certainly many other transition paths with higher energy barriers but they are not important for the system stability. If we had missed such a transition, this would mean that there is a transition path with an energy barrier even smaller than the reversal via the boundary that we have identified.

Using the standard definition for the magnetisation in spherical angles, **s** = (sin*θ*cos*ψ*, sin*θ*sin*ψ*, cos*θ*), the linear interpolation initial state of the magnetisation is obtained through the angles of corresponding spins, between two different images, say **Y**_*i*_ and **Y**_*k*_ with *i* < *k* (we usually use the extreme images, hence *i* = 0 and *k* = *N*). Thus, for every spin *j* ∈ {0, …, *P* − 1} of the system, if we perform *n* interpolations, the interpolated angles (*θ*_*j*_^(*l*)^, *ψ*_*j*_^(*l*)^) of the image **Y**_*l*_, *l* ∈ {*i*, …, *k*}, are

10 $$\begin{array}{ll}{\theta }_{j}^{(l)}= & {\theta }_{j}^{(i)}+\frac{l}{n+1}[{\theta }_{j}^{(k)}-{\theta }_{j}^{(i)}]\\ {\psi }_{j}^{(l)}= & {\psi }_{j}^{(i)}+\frac{l}{n+1}[{\psi }_{j}^{(k)}-{\psi }_{j}^{(i)}]\end{array}$$

Regarding the spring force, Dittrich *et al*.^17^ stated that their results did not require its application when using a variable order and time step method. In our study, the spring force has an influence in the results, affecting the number of iterations necessary for the algorithm converge and to achieve a better equispaced band. An estimation of the spring constant *k* in equation 8 is difficult to compute, because it depends on many factors of the NEBM, such as the size of the system, number of spins, interactions involved or the coordinates system. We performed a series of tests to check optimal values for *k*, and it is usually in a range around 10^2^ to 10^5^. For larger order of magnitudes, the algorithm requires significant computation time, specially when using a small criteria for stopping the algorithm.

We define the convergence of the NEBM as follows. We first calculate the norms of the difference (in corresponding degrees of freedom) between the energy bands of the last NEBM step and the previously computed step. Consequently we scale them by the number of degrees of freedom (spins) per image and finaly compute the maximum of these norms and divide by the last time discretisation given by the integrator^43^. Thus, we say the band converged if this value if smaller than a specified criteria. We usually use a value about 10^−6^.

### Topological charge

For a hexagonally arranged discrete spins lattice, we use a topological charge *Q* defined for discrete lattices, which was proposed by Berg and Lüscher^44^ and, recently, applied by Yin *et al*.^31^ to square arrangements. This is based on taking two spherical triangles per lattice site, where every triangle is defined by three neighbouring spins, covering the whole lattice area. This mapping counts the number of times the spin directions cover a unit sphere. For details, see the Topological charge section in the Supplementary Information.

### Computational simulations

The NEBM atomistic simulations were performed with Fidimag^24^, a finite differences code written in Python and C, that uses Sundials^43^ for integrating the dynamical equations. In addition, Matplotlib^45^, IPython^46^ and the Jupyter notebook^47^ were used for data analysis, and Mayavi^48^ and Povray^49^ for the figures.

## Electronic supplementary material

Video S1

Video S2

Video S3

Video S4

Video S5

Video S6

Supplementary Information
