# Supplementary material for: Thermal stability and topological protection of skyrmions in nanotracks
Source: Sci Rep. 2017 Jun 22;7:4060. doi: 10.1038/s41598-017-03391-8 (PMC5481343; doi:10.1038/s41598-017-03391-8)
Supplement: Supplementary file 7 — Supplementary Information [file 41598_2017_3391_MOESM7_ESM.pdf]

Thermal stability and topological protection of skyrmions in nanotracks (Supplementary Information)

David Cortés-Ortuño,[1,*] Weiwei Wang,[1,2] Marijan Beg,[1] Ryan A. Pepper,[1] Marc-Antonio Bisotti,[1] Rebecca Carey,[1] Mark Vousden,[1] Thomas Kluyver,[1] Ondrej Hovorka,[1] and Hans Fangohr[1,3,†]

[1]*Faculty of Engineering and the Environment, University of Southampton, Southampton, SO17 1BJ, United Kingdom*

[2]*Department of Physics, Ningbo University, Ningbo, 315211, China*

[3]*European XFEL GmbH, Holzkoppel 4, 22869 Schenefeld, Germany*

TEST MODEL

Bessarab et al.[S1] proposed a toy model to simulate a skyrmion on a discrete spins lattice in order to test the Nudged Elastic Band Method (NEBM). The model Hamiltonian reads

$$H = -\sum_{\langle i,j \rangle}^{N} J_{ij}\mathbf{s}_i \cdot \mathbf{s}_j - \sum_{\langle i,j \rangle}^{N} D_{ij}\left[\mathbf{s}_i \times \mathbf{s}_j\right] - \sum_{i}^{N} \mu_i \mathbf{B} \cdot \mathbf{s}_i \tag{1}$$

which has the convention $\langle i, j \rangle$ in the sums, indicating a summation over every spin pair without repetition. The magnetic parameters are: an exchange constant of $J = 10\,\mathrm{meV}$, a DMI constant of $D = 66\,\mathrm{meV}$, a magnetic moment of $\mu_s = 2\mu_B$ and an out of plane magnetic field of 25 T. Accordingly, we reproduced the system with our finite differences code and obtained a good agreement using Geodesic distances to distinguish the images and Cartesian coordinates to describe the spins. The result is shown in Supplementary Fig. S1, where an energy barrier of approximately 40 meV, with respect to the skyrmion energy, was found after a reasonable number of NEBM iterations.

This test model can be reproduced using the code from the repository in Ref. S2.

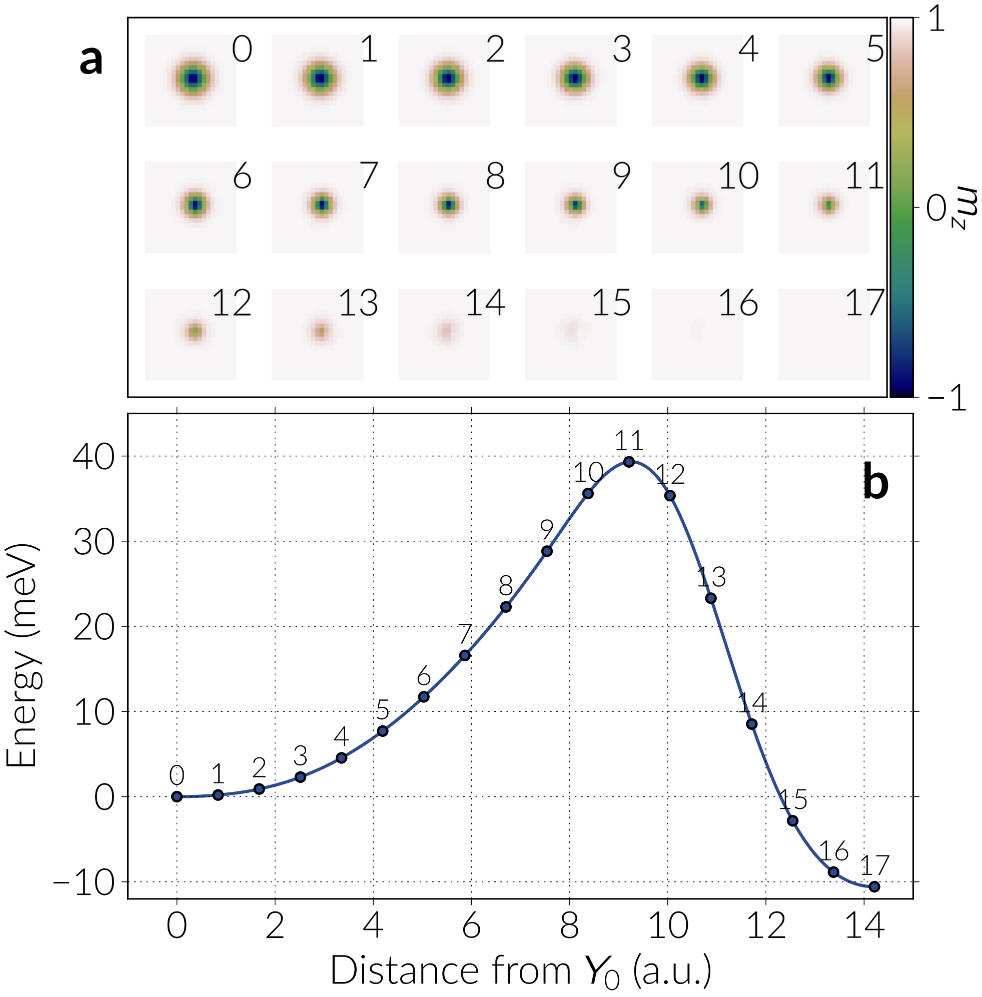

SUPP. FIG. S1. **Energy band of of a skyrmion in a 21x21 spins system**. Spins are arranged in a two dimensional square lattice. Supplementary Figure (b) shows the images of the band depicted in Supplementary Fig. (a), colourised according to the out of plane spin directions magnitude.

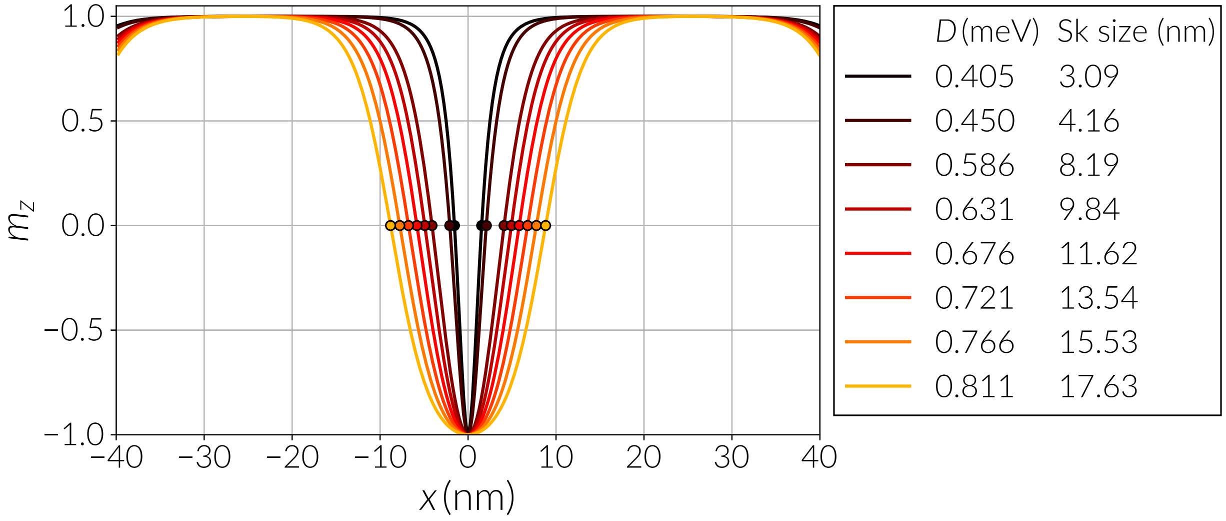

SUPP. FIG. S2. **Skyrmion profile as a function of the DMI magnitude**. For every case we show a continuous line that connects the discrete data points m_z from every lattice site across the nanotrack long side (in the x direction). Small circles indicate points where $m_z = 0$ and using the distance between them we obtain the skyrmion size.

NANOTRACKS

In the main text we specified a range of DMI values to analyse the minimum energy transitions of the skyrmions. Specifically, we analysed skyrmions for DMI values from $D = 0.586\,\text{meV}$ to $0.811\,\text{meV}$ in steps of $0.045\,\text{meV}$. We obtained these skyrmions after relaxing them using the Landau-Lifshitz-Gilbert equation. In Supplementary Fig. S2 we show the skyrmion profile for every case, by plotting the out of plane magnetisation component m_z at the middle and across the long edge of the tracks. Using the convention of defining the skyrmion size as the width within the points where $m_z = 0$, we can see that the skyrmion size increases with larger DMI magnitudes. In addition, there is a small tilting at the boundaries which makes a skyrmion on the system to have a magnitude for the total topological charge, slightly smaller than one (or not perfectly zero for the ferromagnetic state).

Initial states

Given an initial energy band, the Nudged Elastic Band Method (NEBM) will determine the lowest energy transition path between two equilibrium configurations that is accessible

from the initial band. In general, it is difficult to predict an optimal initial guess for the bands, in particular for a system with such a high degree of complexity. In the context of the skyrmion transition, linear interpolations seem a robust starting point and are easily reproducible and applicable to different magnetic systems. A similar technique for initialising the system, where spin directions are interpolated, is using Rodrigues formula[S1].

After exploring the system numerically, we also noticed the major role of the boundaries in the skyrmion annihilation and it was necessary to create an initial path close to this transition, which yields lower energy transition paths. Another way of guessing an initial path would be, for example, to use the dynamics that the system follows when applying spin polarised currents (as Sampaio et al. do in Ref. S3) or magnetic fields (that is not the best choice since the Zeeman energy changes the energy landscape configuration). For the systems of this study, these paths converge to the same results obtained with linear interpolations .

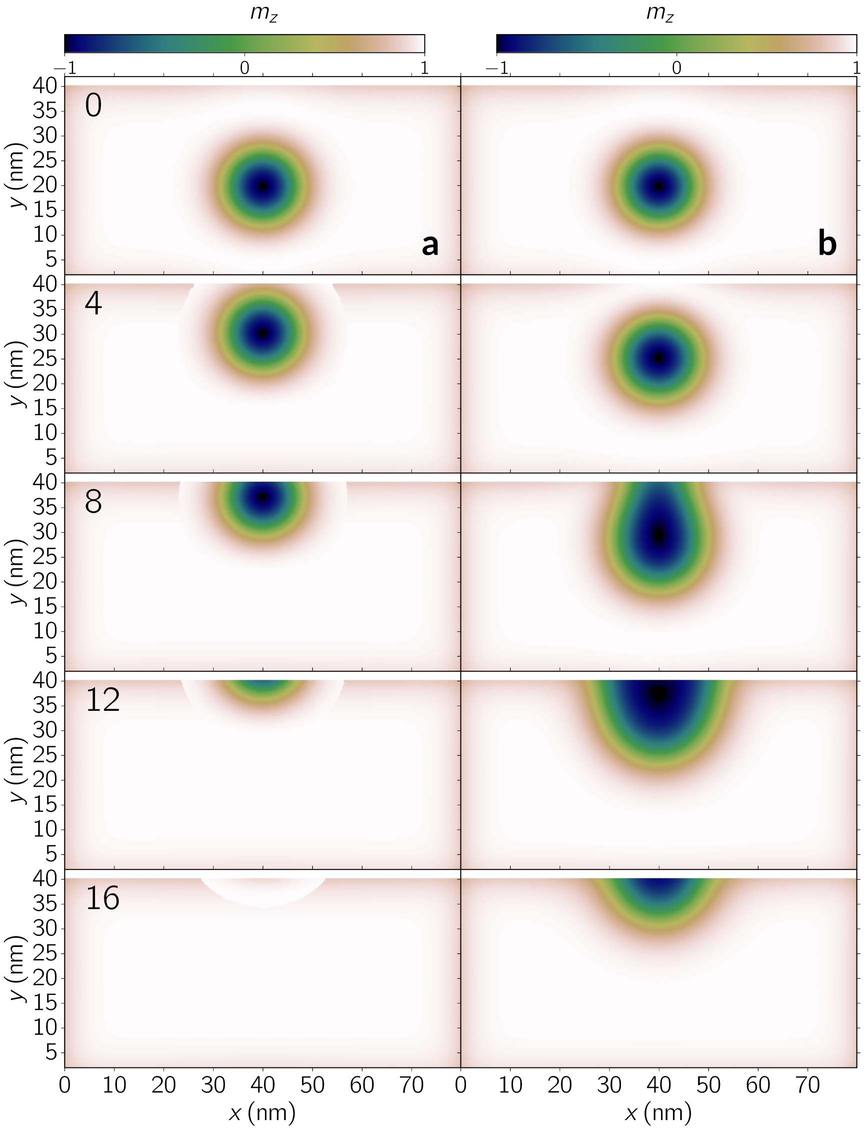

SUPP. FIG. S3. **Initial and final bands for the boundary annihilation energy path**. We use a band of 21 images based on a system with a DMI magnitude of $D = 0.721\,\mathrm{meV}$. Numbers on the top left of every row indicate the image number in the band. (a) Initial state for the NEBM algorithm. (b) Skyrmion annihilation through a boundary obtained after relaxing the band of column (a) with the NEBM.

Energy band images

In our study we found three main energy paths for the skyrmion annihilation. For clarity, we provide here more detailed versions of the bands for every transition. Firstly, in Supplementary Fig. S3 we show the skyrmion annihilation through the boundary of the nanotrack, when using a DMI magnitude of $D = 0.721\,\mathrm{meV}$ and a band made of 21 images. In the first column of the figure, *i.e.* Supplementary Fig. S3a, we plot the initial state for the NEBM algorithm, which we manually generate, thus we can see that the skyrmion boundaries are not perfectly defined, specially when approaching the stripe edge. The numbers at the top left of Supplementary Fig. S3 indicate the image number. Consequently, we apply the NEBM to this band and obtain the sequence shown in Supplementary Fig. S3b, where helicoidal structures when the skyrmion is destroyed at the boundary are well defined, and the band has overall reduced its energy. Interestingly, the helicoidal structures are similar to those shown in in Ref. S3, when skyrmions are not successfully generated with spin polarised currents.

Similarly, in Supplementary Fig. S4 we show the skyrmion destruction transition in three different stages, when using a DMI magnitude of $D = 0.721\,\mathrm{meV}$ and a band made of 27 images. In Supplementary Fig. S4a we depict the initial band when using linear interpolations on the spherical angles for the magnetisation field. The numbers on the top left indicate the image number in the band. Consequently, Supplementary Fig. S4b illustrates the sequence after relaxing the initial state with the NEBM, where the final state is the skyrmion destruction by a singularity that resembles a Bloch point, as discussed in the main text. Finally, the last column, shown in Supplementary Fig. S4c, is the band of Supplementary Fig. S4b relaxed with the climbing image NEBM, which we applied to the largest energy point of the band. For this case we clearly see the skyrmion collapse path.

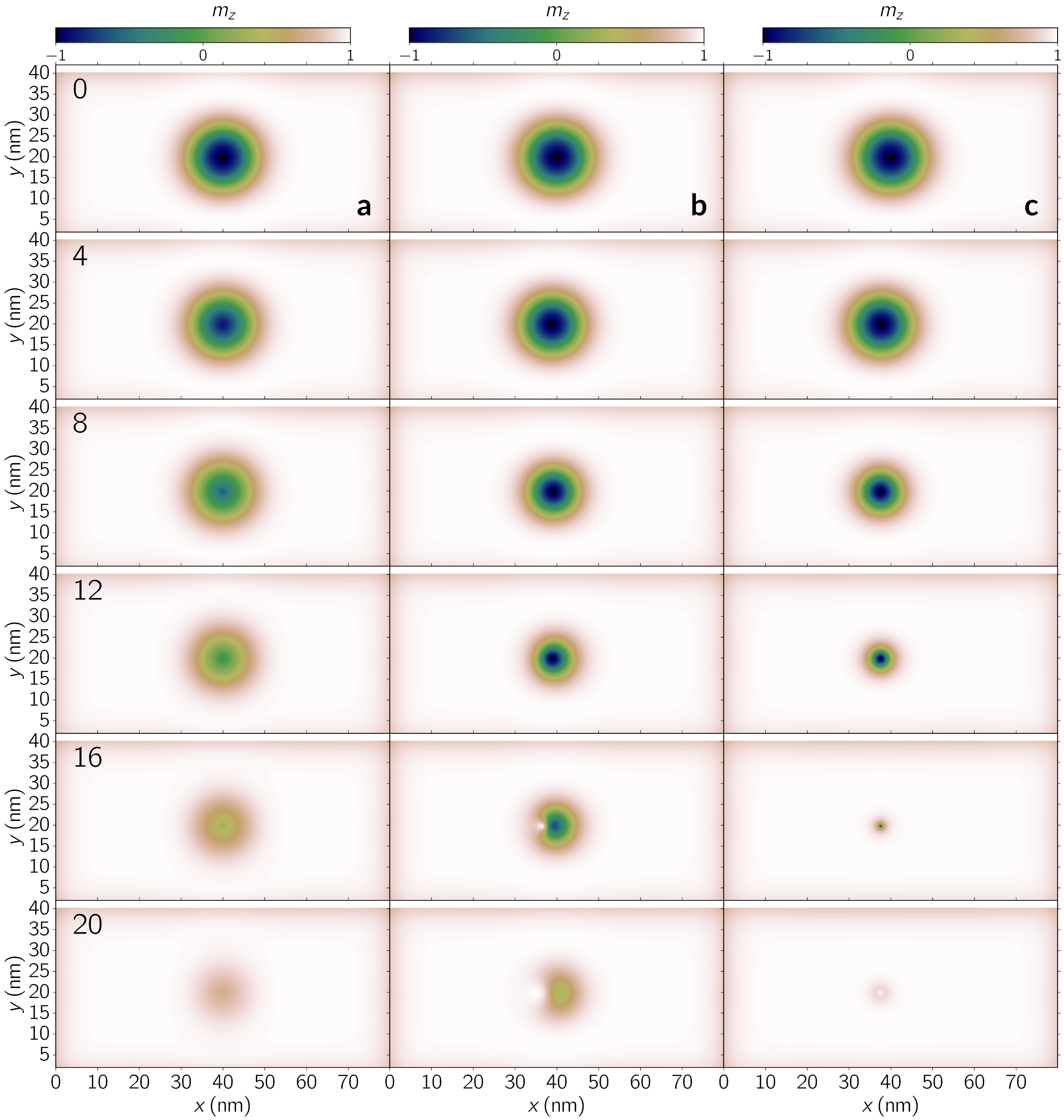

SUPP. FIG. S4. **Skyrmion destruction energy paths in three steps**. We use a band of 27 images based on a system with a DMI magnitude of $D = 0.721\,\mathrm{meV}$. Numbers on the top left of every row indicate the image number in the band. **(a)** Initial state obtained using linear interpolations. **(b)** Skyrmion destruction mediated by a singularity, after relaxing the band of column (a) with the NEBM. **(c)** Skyrmion collapse path, after relaxing the band of column (b) with the climbing image NEBM.

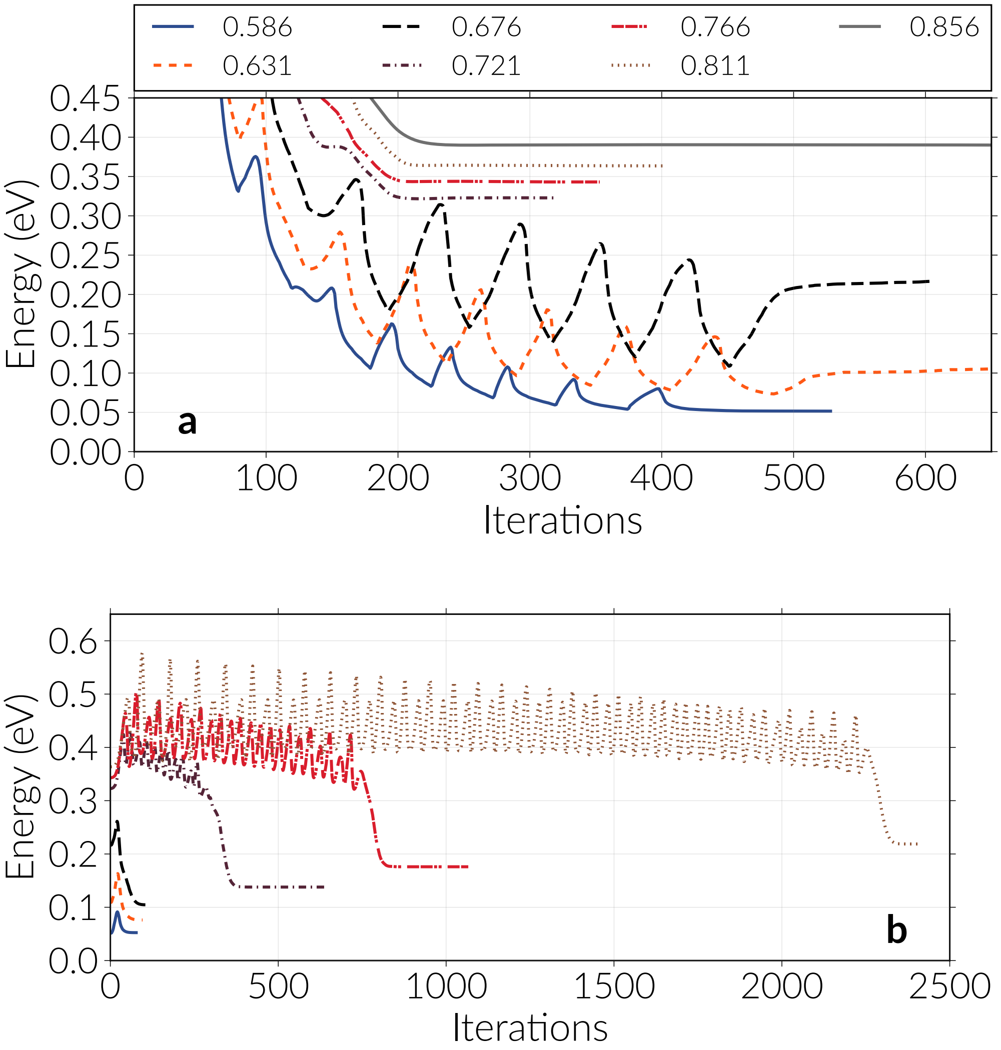

SUPP. FIG. S5. **Evolution of the maximum energy of the bands for the skyrmion destruction processes**. The evolution is computed with respect to the number of iterations of the NEBM relaxation equation (see Methods in the main text) and we show this process for different DMI values. The evolution is calculated using the images with largest energy in the bands. These images can change since they are allowed to move during the iterations. **(a)** Evolution using the NEBM and starting with linear interpolations for the initial bands. **(b)** Evolution using the climbing image NEBM, applied to the bands of (a) after relaxation (last NEBM step).

Skyrmion destruction evolution

According to our implementation of the NEBM, we checked how the maximum energy of the band evolves according to the iterations of the algorithm. We compute this magnitude

using the image with the largest energy in the band, which can change since images move as the NEBM evolves. In Supplementary Fig. S5 we show this evolution for the case of the skyrmion destruction discussed in the main text. In particular, Supplementary Fig. S5a refers to the skyrmion destruction where the bands for $D = 0.721\,\text{meV}$ and above relax to the singularity mediated skyrmion destruction, and for magnitudes of $D = 0.676\,\text{meV}$ and below, the bands relax to the skyrmion collapse. We can observe that for the smaller DMI values, the maximum energy value oscillates before reaching convergence and this is due to the movement of images around the saddle point (where the last spins defining the skyrmion center flip) whose energy fluctuate dramatically when crossing this region.

Moreover, Supplementary Fig. S5b depicts the evolution of the climbing image NEBM technique applied to the relaxed bands of Supplementary Fig. S5a and all the bands relaxed towards the skyrmion collapse. Bands with $D = 0.721\,\text{meV}$ and above take a long time to relax, specially when the DMI gets stronger. As we specified in the main text, we believe this is due to the rough shape of the energy landscape around the saddle point.

ENERGY BARRIERS FOR WEAK DMI

We have analysed the energy barriers for DMI magnitudes in the range 0.586 to 0.811 meV. From Supplementary Fig. 5 in the manuscript, the tendency is that the skyrmion collapse curve decreases almost linearly in this range and extrapolating the curve it seems that at some small D value it will cross the boundary annihilation curve. We then computed the values of the energy barriers for DMI magnitudes weaker than 0.586 meV. Decreasing in steps of 0.045 meV, we could not stabilise a skyrmion for D smaller than 0.405 meV, probably due to the strong exchange and anisotropy which are kept fixed. For this small DMI magnitude a skyrmion is only 3.09 nm wide.

We show in Supplementary Fig. S6 the energy barriers computed for the whole range of DMI values in the main study and down to 0.405 meV. In this plot, we show both the energy barriers obtained using the image with the largest image in a band, which we call NEBM curve, and the energy barriers obtained after performing a polynomial interpolation on the bands, named Interpolation curve. In general, the interpolation gives optimal results for the boundary annihilation, which can be seen from Supplementary Fig. 4 in the manuscript. For the skyrmion collapse the polynomial approximation appears to agree well with the largest

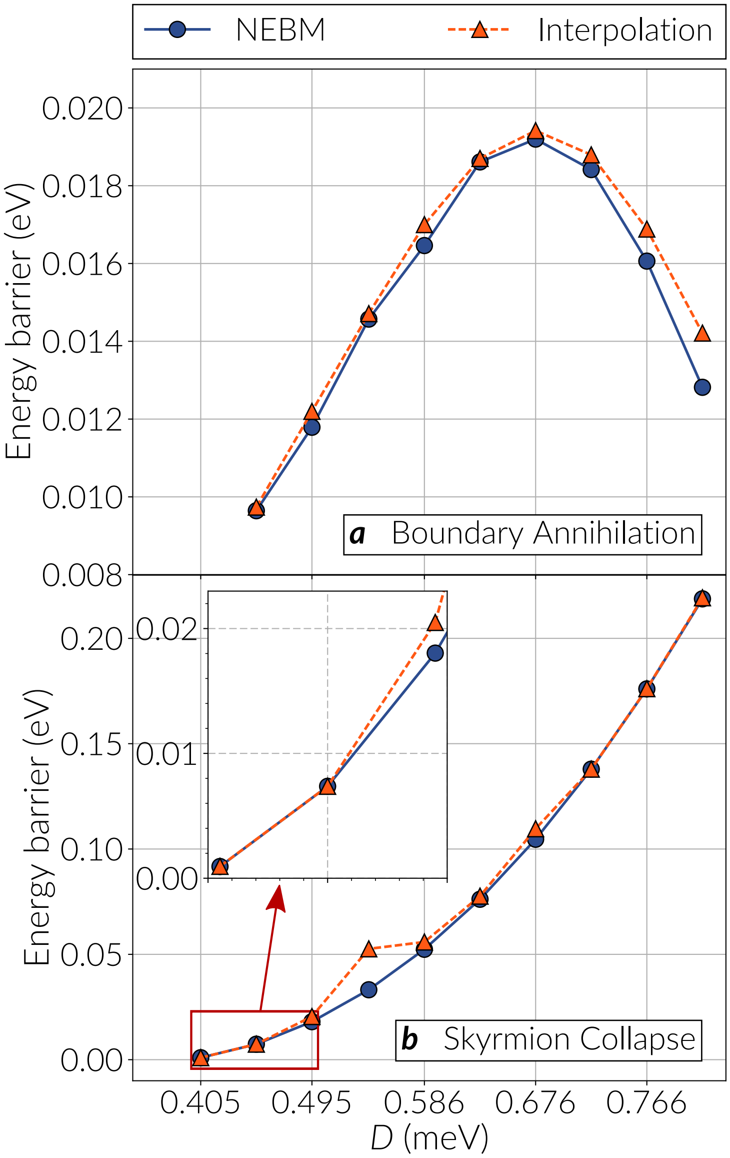

SUPP. FIG. S6. **Calculation of the energy barriers for an extended range of DMI magnitudes**. The NEBM curves show the energy barriers using the image with largest energy in the energy bands and the Interpolation curve are barriers computed when using a cubic polynomial interpolation on the bands. The energy barriers are calculated with respect to the skyrmion energy. The skyrmion cannot be stabilised below 0.405 meV. For $D = 0.541\,\text{meV}$ the polynomial interpolation overestimates the value of the energy barrier. The inset shows the skyrmion collapse energy barriers for the three smallest DMI magnitudes.

point of the band in most cases, but sometimes it can slightly overestimate the energy barrier when there is no good resolution around the saddle point, which is given by the reversal of the spins at the skyrmion nucleus. In particular, this happens for $D = 0.541$ meV, which is shown in Supplementary Fig. S6b. Nevertheless, the quadratic tendency is clear when using the points from the NEBM curve. Interestingly, when comparing the inset of Supplementary Fig. S6b with Supplementary Fig. S6a at $D = 0.450$ meV, where the skyrmion is only 4.16 nm wide, the energy barrier of the skyrmion collapse is slightly smaller. Decreasing the DMI even further we could not obtain a clear value for the boundary annihilation, however in this range the skyrmion collapse barrier is smaller than 0.002 meV.

TOPOLOGICAL CHARGE

The topological charge gives us an idea about the contribution of the spin directions to the mapping of the magnetisation field into a unit sphere. This helps us to characterise non trivial magnetic structures, such as the Bloch point like structure we observed in our simulations. From our results, a full mapping of the spins around this topological singularity would not be optimal, since the full structure usually lies between two images around a saddle point. Nevertheless, it is still possible to observe large variations of the topological charge density when the singularity starts to appear, allowing us to distinguish it in the spin field.

In micromagnetics, instead of calculating a topological charge, it is standard to compute the so called skyrmion number, which requires the calculation of the derivatives of the magnetization field in a two dimensional plane. For a discrete spins lattice we use the topological charge definition rather than discretising the derivatives along the 2D-lattice directions (since, for the sake of numerical calculations, we have to anyways discretise the continuum micromagnetic mesh), as Rohart et al. mention in S4. As we specified in the main text, we base our calculation on Ref. S5 and S6, where we have extended the definition to hexagonally arranged spin lattices. This calculation is based on computing the spherical angle spanned by triplets of neighbouring spins per every lattice. Accordingly, the total topological charge is defined as

$$Q = \sum_{\mu} q_{\mu} = \sum_{\mu} \frac{1}{4\pi} \left(\Omega_{\mu,1,2} + \Omega_{\mu,3,4} \right) \tag{2}$$

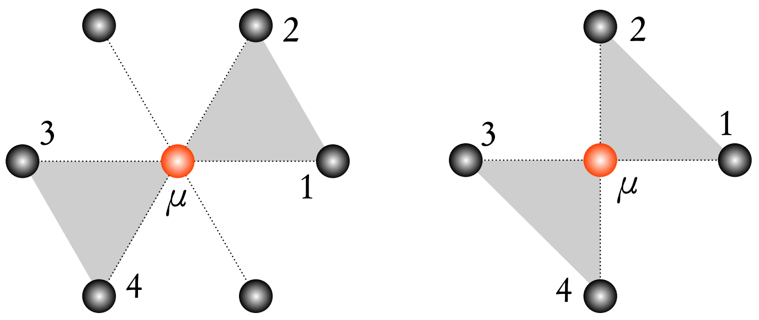

SUPP. FIG. S7. **Triangles of neighbouring spins per lattice site**. Sites are labelled as μ to define a topological charge or skyrmion number for discrete hexagonal (left image) and square (right image) lattices.

The spherical angle at the μ-th site $\Omega_{\mu,1,2}$, is defined as with respect to the neighbouring spins $\mathbf{s}_1$, $\mathbf{s}_2$ as

$$\exp\left(\frac{1}{2}i\Omega_{\mu}\right) = \frac{1}{\rho}\left[1 + \mathbf{s}_{\mu}\cdot\mathbf{s}_1 + \mathbf{s}_1\cdot\mathbf{s}_2 + \mathbf{s}_2\cdot\mathbf{s}_{\mu} + i\mathbf{s}_{\mu}\cdot(\mathbf{s}_1\cdot\mathbf{s}_2)\right] \tag{3}$$

with

$$\rho = \left[2\left(1 + \mathbf{s}_{\mu}\cdot\mathbf{s}_1\right)\left(1 + \mathbf{s}_1\cdot\mathbf{s}_2\right)\left(1 + \mathbf{s}_2\cdot\mathbf{s}_{\mu}\right)\right] \tag{4}$$

Similarly for the μ-th site $\Omega_{\mu,3,4}$. For a square and hexagonal arrangement, the triangles specified per lattice site are depicted in Supplementary Fig. S7 (it would be equivalent to use the opposite triangles that also cover a unit cell). It is worth noticing that Berg and Lüscher[S5] originally introduced a sign for the exponential in equation 3 but we assume the convention that triangles of spins are taken in the counter-clock wise direction, as Yin et al. do in Ref. S6. Moreover, this angle is not completely defined for the special configurations where $\mathbf{s}_{\mu}\cdot(\mathbf{s}_1\cdot\mathbf{s}_2) = 0$ and $1 + \mathbf{s}_{\mu}\cdot\mathbf{s}_1 + \mathbf{s}_1\cdot\mathbf{s}_2 + \mathbf{s}_2\cdot\mathbf{s}_{\mu}$. For these cases, the exponential falls in a branch cut in the complex plane[S5,S6], which implies a change in the topological charge that can be related to the condition where a skyrmion is created or collapses.

The definition of the density charge only considers two triangles around a lattice site, missing the contribution from the other neighbours, but this is necessary to avoid double counting of areas when summing up all the triangles contributions. For a more complete picture of the topological charge per lattice site, we could also take into account the rest of the nearest neighbours.

SUPPLEMENTARY VIDEOS

SUPPLEMENTARY VIDEO S1. **Relaxation of an energy band for the skyrmion collapse**. The animation shows the evolution of the NEBM applied to a nanotrack with $D = 0.676\,\text{meV}$. The initial state at the 0th step is a linear interpolation between the skyrmion (image at the left extreme of the band) and the ferromagnetic (right extreme) states. The band at the final steps represents the skyrmion collapse transition, where the image with the largest energy is the saddle point. Around this image, the corresponding magnetic configurations characterise the reversion of the spins at the skyrmion core.

SUPPLEMENTARY VIDEO S2. **Relaxation of an energy band for the singularity mediated skyrmion destruction**. The animation shows the evolution of the NEBM applied to a nanotrack with $D = 0.721\,\text{meV}$. The initial state at the 0th step is a linear interpolation between the skyrmion (image at the left extreme of the band) and the ferromagnetic (right extreme) states. The band at the final steps represents the skyrmion destruction via a Bloch point like singularity. The image with the largest energy is the saddle point and around this image, the corresponding magnetic configurations characterise the emergence of the magnetic singularity.

SUPPLEMENTARY VIDEO S3. **Climbing image NEBM applied to the skyrmion destruction by a singularity**. The animation shows the evolution of the climbing image technique applied to the image with largest energy (saddle point) in the band from the last step of Video 2, which is the skyrmion annihilation mediated by a singularity. This system has DMI of $D = 0.721\,\text{meV}$. In the video, the climbing image is labeled during the algorithm evolution. At the end of the relaxation, the energy band converges to an energy band representing a skyrmion collapse, which has a smaller energy barrier.

SUPPLEMENTARY VIDEO S4. **Annihilation of a skyrmion through a boundary**. This animation shows the first images of an energy band representing the skyrmion annihilation mediated by a boundary. The sequence is restricted to a region around a boundary of the nanotrack where the skyrmion destruction occurs. The animation was obtained from

the NEBM simulation applied to a nanotrack with $D = 0.721\,\mathrm{meV}$ and the top left numbers are the image numbers in the band.

SUPPLEMENTARY VIDEO S5. **Skyrmion collapse**. This animation shows the first images of an energy band representing the skyrmion collapse transition. The sequence is restricted to a region at the centre of the nanotrack where the skyrmion destruction occurs and spins are only shown for some lattice sites. The animation was obtained from the climbing image NEBM simulation applied to a nanotrack with $D = 0.721\,\mathrm{meV}$ and the top left numbers are the image numbers in the band.

SUPPLEMENTARY VIDEO S6. **Skyrmion destruction mediated by a singularity**. This animation shows the first images of an energy band representing the skyrmion annihilation by a Bloch point like singularity. The sequence is restricted to a region at the centre of the nanotrack, where the skyrmion destruction occurs, and spins are only shown for some lattice sites. The animation was obtained from the NEBM simulation applied to a nanotrack with $D = 0.721\,\mathrm{meV}$ and the top left numbers are the image numbers in the band.

---

[*] d.i.cortes@soton.ac.uk

[†] h.fangohr@soton.ac.uk

[S1] Bessarab, P. F., Uzdin, V. M. & Jónsson, H. Method for finding mechanism and activation energy of magnetic transitions, applied to skyrmion and antivortex annihilation. *Comput. Phys. Commun.* **196**, 1–37 (2015).

[S2] Cortés-Ortuño, D. & Fangohr, H. Test system for nudged elastic band method in nanoscale magnetism. Zenodo doi: `https://doi.org/10.5281/zenodo.167870` (2016).

[S3] Sampaio, J., Cros, V., Rohart, S., Thiaville, a. & Fert, A. Nucleation, stability and current-induced motion of isolated magnetic skyrmions in nanostructures. *Nat. Nanotechnol.* **8**, 839–44 (2013).

[S4] Rohart, S., Miltat, J. & Thiaville, A. Path to collapse for an isolated Néel skyrmion. *Phys. Rev. B* **93**, 214412 (2016).

[S5] Berg, B. & Lüscher, M. Definition and statistical distributions of a topological number in the lattice $O(3)\,\sigma$–model. *Nuclear Physics, Section B* **190**, 412–424 (1981).

[S6] Yin, G. *et al.* Topological charge analysis of ultrafast single skyrmion creation. *Phys. Rev. B* **93**, 174403 (2016).
